# Evaluation of Itraconazole as a repurposed small molecule inhibitor of EMT in breast cancer: a molecular docking and dynamics study

**DOI:** 10.1007/s00894-026-06852-y

**Published:** 2026-07-18

**Authors:** Prasanna Kumar Reddy Gayam, Aniruddha Murahar Kulkarni, Jesil Mathew Aranjani

**Affiliations:** 1 Department of Pharmaceutical Biotechnology, Manipal College of Pharmaceutical Sciences, Manipal Academy of Higher Education, Manipal, India; 2 Present Address: Department of Developmental Biology and Genetics, Indian Institute of Science, Bangalore, 560012 Karnataka India

**Keywords:** Itraconazole, Epithelial‒mesenchymal transition, Breast cancer, Metastasis, Molecular dynamics, Drug repurposing

## Abstract

**Context:**

Epithelial-Mesenchymal Transition (EMT) is a critical driver of metastasis and drug resistance in breast cancer. Repurposing FDA-approved drugs offers a rapid strategy to target EMT pathways. This study evaluates Itraconazole, an antifungal agent with reported anti-cancer properties, as a potential multi-target inhibitor of the EMT signaling network. We investigated the binding mechanism and selectivity of Itraconazole against four key EMT regulators: Smoothened (SMO), TGF-βR1, EGFR, and GLI1. Our results identify SMO as the primary target, exhibiting high binding affinity and thermodynamic stability. Conversely, EGFR and GLI1 displayed significant structural instability, indicating a lack of direct inhibition. The study provides atomic-level structural evidence supporting the repurposing of Itraconazole as a selective, combinatorial therapeutic agent to target EMT-driven metastasis.

**Methods:**

Induced Fit Docking (IFD) was performed using the Schrödinger IFD protocol and the Glide XP scoring function within the Schrödinger Maestro suite. The dynamic stability of the protein–ligand complexes was evaluated using 500 ns molecular dynamics (MD) simulations with the Desmond software and the OPLS4 force field. To differentiate false positives and assess pose stability under enhanced sampling, Binding Pose Metadynamics (BPMD) was utilized. Thermodynamic binding free energies were calculated using the Thermal MM/GBSA method. All simulations were analyzed using the Simulation Interaction Diagram and trajectory clustering tools within Schrödinger.

## Introduction

According to the most recent available statistics, since the year 2020, the number of female patients with breast cancer has peaked at approximately 2.3 million cases globally, and the disease has resulted in the mortality of 685,000 women in the same year [1]. Risk factors for this disease include age, reproductive factors (early menarche, late menopause, lower exposure to breastfeeding), nonreproductive factors (obesity, alcohol consumption, smoking) and genetic factors (mutations in BRCA1/2 genes) [2, 3]. The substantial mortality rate is attributed to metastases in distant organs rather than the primary tumor itself [4, 5]. Metastatic progression occurs through the invasion of primary tumor cells into the surrounding tissue, entry into the blood or lymphatic circulation, extravasation at a distant site and the development of metastatic growth. For the cascade to succeed, few modifications in the cell phenotype are needed, such as lowered cell‒cell adhesion, the ability of the extracellular matrix (ECM) to degrade, and improved migratory potential. Substantial evidence indicates that these changes are caused by epithelial‒mesenchymal transition (EMT) [5].

EMT refers to the series of events in which the phenotype of an epithelial cell shifts toward that of a mesenchymal cell. The physiological contexts in which the EMT occurs include embryogenesis and wound healing. However, cancer progression is a pathological consequence of EMT [6]. Various degrees of EMT have been observed in cancer cells, with a hybrid state possessing the highest metastatic potential [7]. Epithelial markers such as E-cadherin, claudins and the miR-200 family are downregulated, whereas mesenchymal markers such as vimentin, N-cadherin, matrix metalloproteases (MMPs), and CD44 are upregulated [6]. EMT is governed by a variety of ligands and signaling pathways, which eventually activate pro-EMT transcription factors such as Snail, Slug, Twist, FOXC1, and FOXC2, which are responsible for the differential expression of the aforementioned biomarkers. These pathways include the TGF-β, Notch, Wnt, Hedgehog, and cytokine signaling pathways [8].

In this study, we performed a comparative computational analysis to profile the binding and selectivity of Itraconazole against four key proteins implicated in EMT in breast cancer: TGF-βRI, Smoothened (SMO), GLI1, and Epidermal Growth Factor Receptor (EGFR). Itraconazole was chosen as the molecule of choice based on an earlier review [9], which summarized the various findings pertaining to EMT inhibitory activity of Itraconazole. This study aims to elucidate the molecular basis for Itraconazole's selectivity, providing a structural rationale for its known anti-Hedgehog activity while exploring its potential as a novel multi-target EMT inhibitor. TGF-βRI is recruited and activated upon the binding of the TGF-β ligand to the TGF-βRIII receptor via Smad-dependent and Smad-independent pathways, pro-EMT transcription factors are upregulated, thereby leading to the EMT phenotype [8]. Smoothened (SMO) and GLI family members are downstream effectors of the Hedgehog pathway, which are responsible for the expression of multidrug resistance protein-1 (MDR-1) and vascular endothelial growth factor receptor 2 (VEGFR2), which play a role in drug resistance and angiogenesis, respectively [10–12]. Epidermal growth factor receptor (EGFR) is also implicated, especially in triple-negative breast cancer (TNBC), to confer properties of EMT, invasion, metastasis and poor clinical outcomes [13, 14].

## Results

### Induced fit docking

**Fig. 1:**
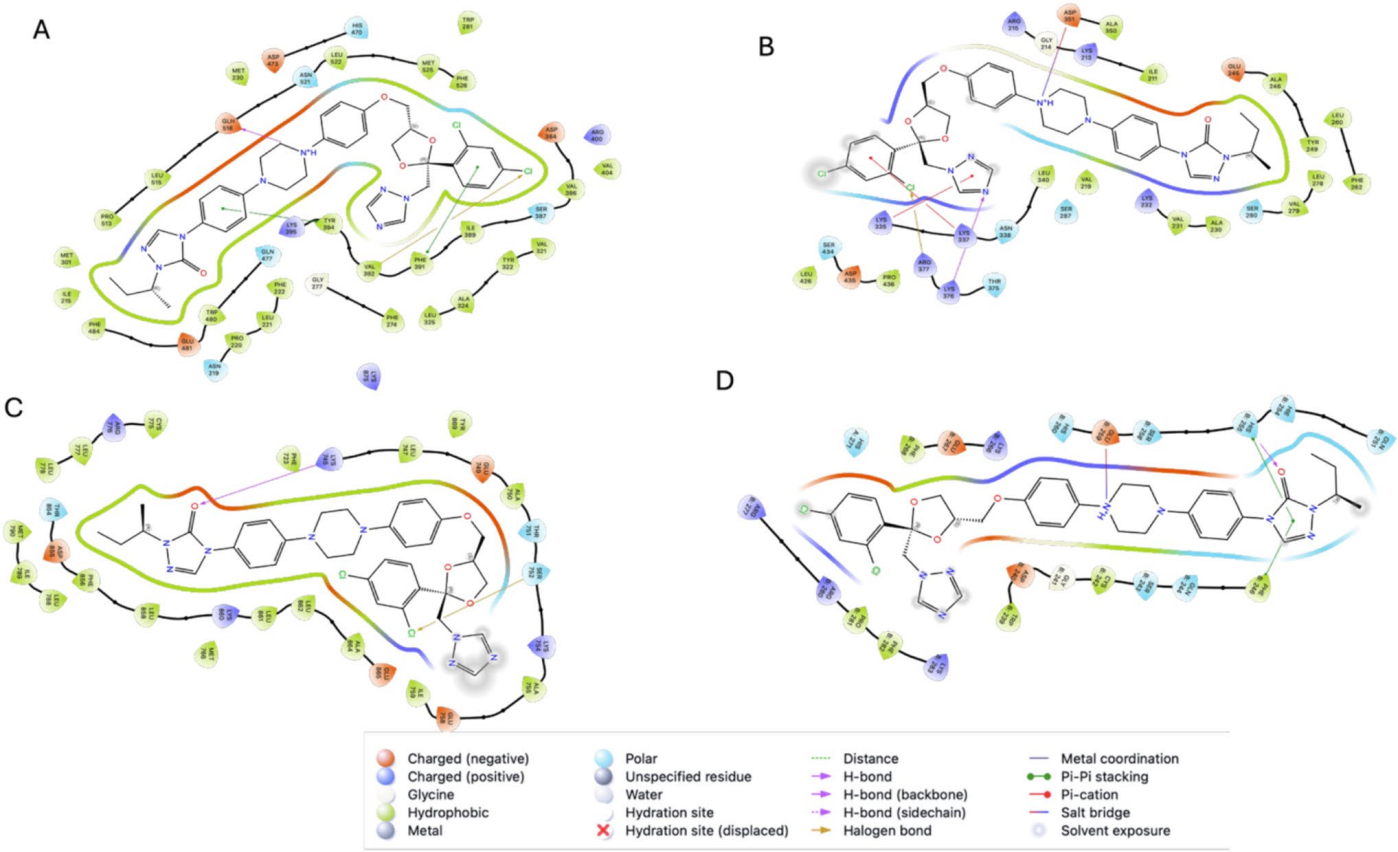
Docking interactions of Itraconazole with the selected proteins, along with the legend; **A**) Smoothened; **B**) TGF-βR1; **C**) EGFR; **D**) GLI1

The Induced Fit Docking (IFD) results demonstrated that Itraconazole exhibited the highest predicted binding affinity for the Smoothened (SMO) protein, achieving a highly favorable docking score of − 15.405 kcal/mol. This strong initial binding pose was characterized primarily by hydrogen bonds with Arg 400 and Asn 219, hydrophobic interactions with Phe 391 and Tyr 394, and water-mediated interactions involving His 470 and Ser 385 (Fig. 1A, Table 1). The second most favorable initial interaction was observed with EGFR, yielding a score of − 12.998 kcal/mol, primarily involving a hydrogen bond to the crucial hinge residue Lys 745 and hydrophobic interactions with Leu 718 and Phe 856 (Fig. 1C). Conversely, TGF-βR1 recorded a less favorable score of − 6.585 kcal/mol, involving hydrogen bonds with Asn 456 and hydrophobic interactions with Ile 454 and Ala 477 in the kinase hinge region (Fig. 1B). GLI1 showed the lowest affinity with a score of − 5.837 kcal/mol, characterized by hydrogen bonds to Gln 244 and Lys 283 within the zinc finger domain (Fig. 1D).

**Table 1:** Itraconazole-protein docking score and interacting amino acids

Sr no	Protein	Docking score (kcal/mol)	Interacting amino acids
1	Smoothened	−15.405	Asparagine 521 & 219 Phenylalanine 391 Tyrosine 394 Serine 385 Arginine 400 Histidine 470
2	TGF-βR1	−6.585	Asparagine 456 Isoleucine 454 Tyrosine 476 Alanine 477
3	EGFR	−12.998	Lysine 745
4	GLI1	−5.837	Histidine 255 Lysine 283 & 266 Glutamine 244

**Fig. 2:**
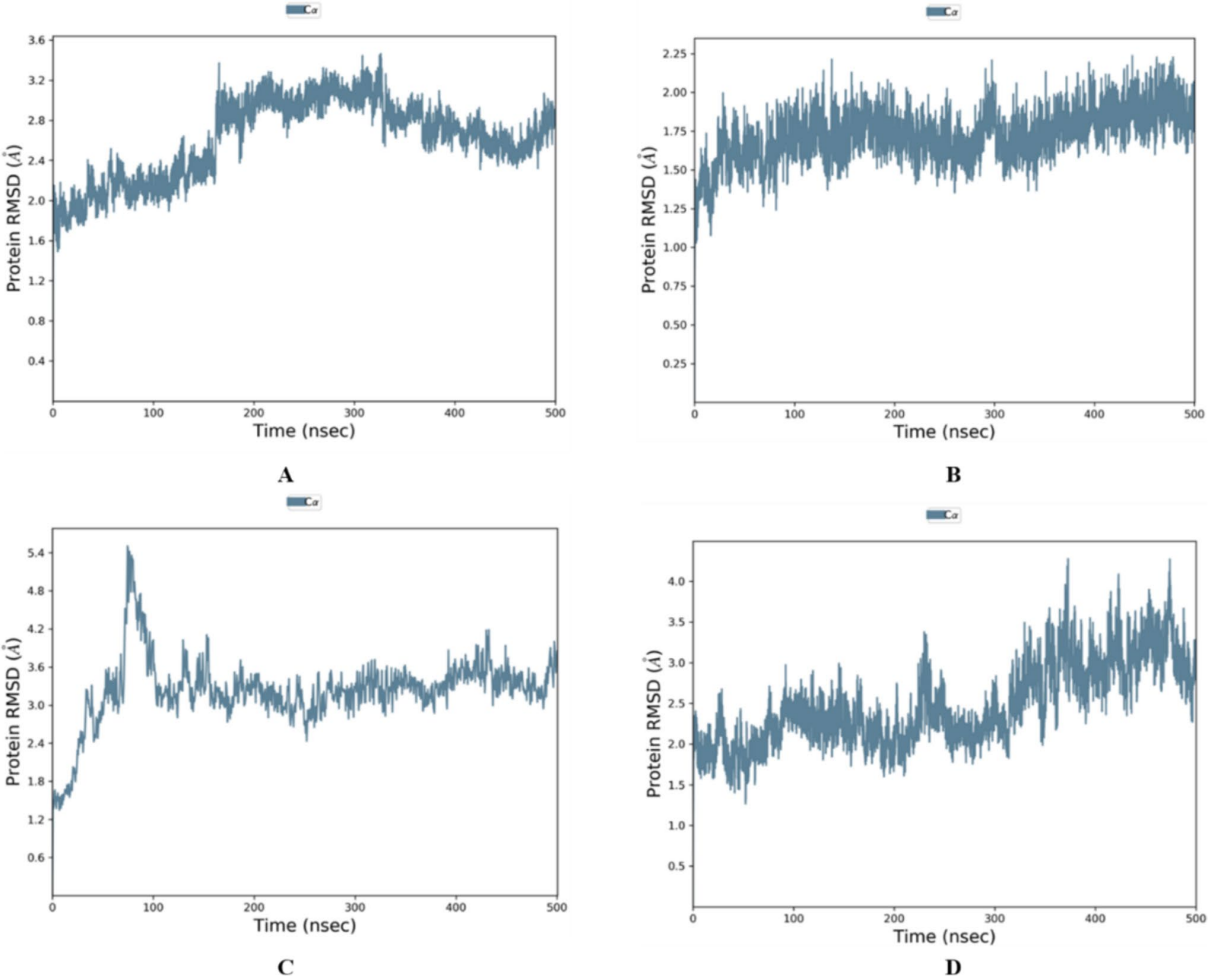
RMSD plots of Itraconazole against corresponding proteins: **A**) Smoothened **B**) TGF-βR1, **C**) EGFR, and **D**) GLI1

To further contextualize Itraconazole's binding predictions, we performed a comparative IFD analysis against known, potent inhibitors for each target (Table 2). This benchmarking provides valuable context for interpreting the relative scores. For SMO, Itraconazole achieved an IFD score (−15.405 kcal/mol) superior to that of the FDA-approved inhibitor Vismodegib (−11.908 kcal/mol) when docked into the same pocket. This strong computational prediction aligns well with literature reports of Itraconazole's potent anti-Hedgehog activity (experimental IC_50_ ≈ 800 nM [15]), reinforcing its status as a high-affinity SMO ligand. Conversely, Itraconazole's score against TGF-βR1 (−6.585 kcal/mol) was significantly weaker than that of the known inhibitor Galunisertib (−11.082 kcal/mol), computationally supporting our assessment of TGF-βR1 as a secondary, lower-affinity target. Interestingly, for EGFR, Itraconazole's docking score (−12.998 kcal/mol) was nearly identical to the potent inhibitor Gefitinib (−13.065 kcal/mol). However, this initial favorable docking pose proved highly unstable during MD simulations (Fig. 2C), unlike the stable complexes formed by established EGFR inhibitors. This highlights the crucial role of MD in assessing dynamic stability, which static docking alone cannot capture, and confirms EGFR is unlikely to be a durable target for Itraconazole. It is further noted that PDB 6DUK captures EGFR in the inactive αC-helix-out conformation, which uniquely exposes the allosteric binding pocket targeted by inhibitors such as EAI045 [16]. The use of this structure was therefore deliberate, allowing exploration of whether Itraconazole could engage EGFR through an allosteric mechanism analogous to its established allosteric inhibition of SMO. The MD-observed instability of this complex indicates that productive allosteric engagement does not occur under the conditions studied.

**Table 2:** Comparative induced fit docking scores between Itraconazole and known inhibitors of corresponding targets

Protein	Ligand	IFD docking score (kcal/mol)
SMO	Itraconazole	−15.405
SMO	Vismodegib	−11.908
TGF-βR1	Itraconazole	−6.585
TGF-βR1	Galunisertib	−11.082
EGFR	Itraconazole	−12.998
EGFR	Gefitinib	−13.065

### Molecular dynamics and interaction analysis

The Molecular Dynamics (MD) simulations were performed for 500 ns to assess the dynamic stability of the protein–ligand complexes and characterize the interaction profiles between Itraconazole and the four target proteins. The overall stability of the protein structures (C-alpha RMSD) was confirmed to remain within acceptable limits throughout the simulations (data not shown), validating the simulation setup and allowing focus on the protein backbone dynamics relative to ligand binding and the specific ligand–protein contacts.

### Protein RMSD analysis

The Root Mean Square Deviation (RMSD) plots for the protein backbone C-alpha atoms (Fig. 2), which reflect the stability of the protein structure upon ligand binding, varied significantly across the complexes, providing key insights into binding viability. The TGF-βR1 complex (B) exhibited the most stable profile, with RMSD values consistently remaining within a narrow range of 1.0 Å to 2.25 Å for the entire 500 ns duration, indicating a highly stable protein conformation upon Itraconazole binding. The Smoothened (SMO) complex (A) showed moderate stability, characterized by a gradual increase in RMSD during the first 250 ns, likely reflecting an 'induced fit' conformational adjustment, before stabilizing around a reasonable value of 3.0 Å for the remainder of the simulation. In stark contrast, the EGFR complex (C) demonstrated significant structural instability, particularly during the initial 100 ns, where the RMSD peaked dramatically around 5.4 Å. Although it later settled into a higher RMSD range (3.0 Å to 4.0 Å), this initial large deviation suggests a poor and potentially non-physical interaction. Similarly, the GLI1 complex (D) exhibited a transient conformational change, with RMSD values reaching approximately 4.2 Å after 350 ns before recovering to baseline values of approximately 2 Å by the end of the trajectory, suggesting conformational flexibility of the complex rather than irreversible destabilization.

### Contact histogram analysis

**Fig. 3:**
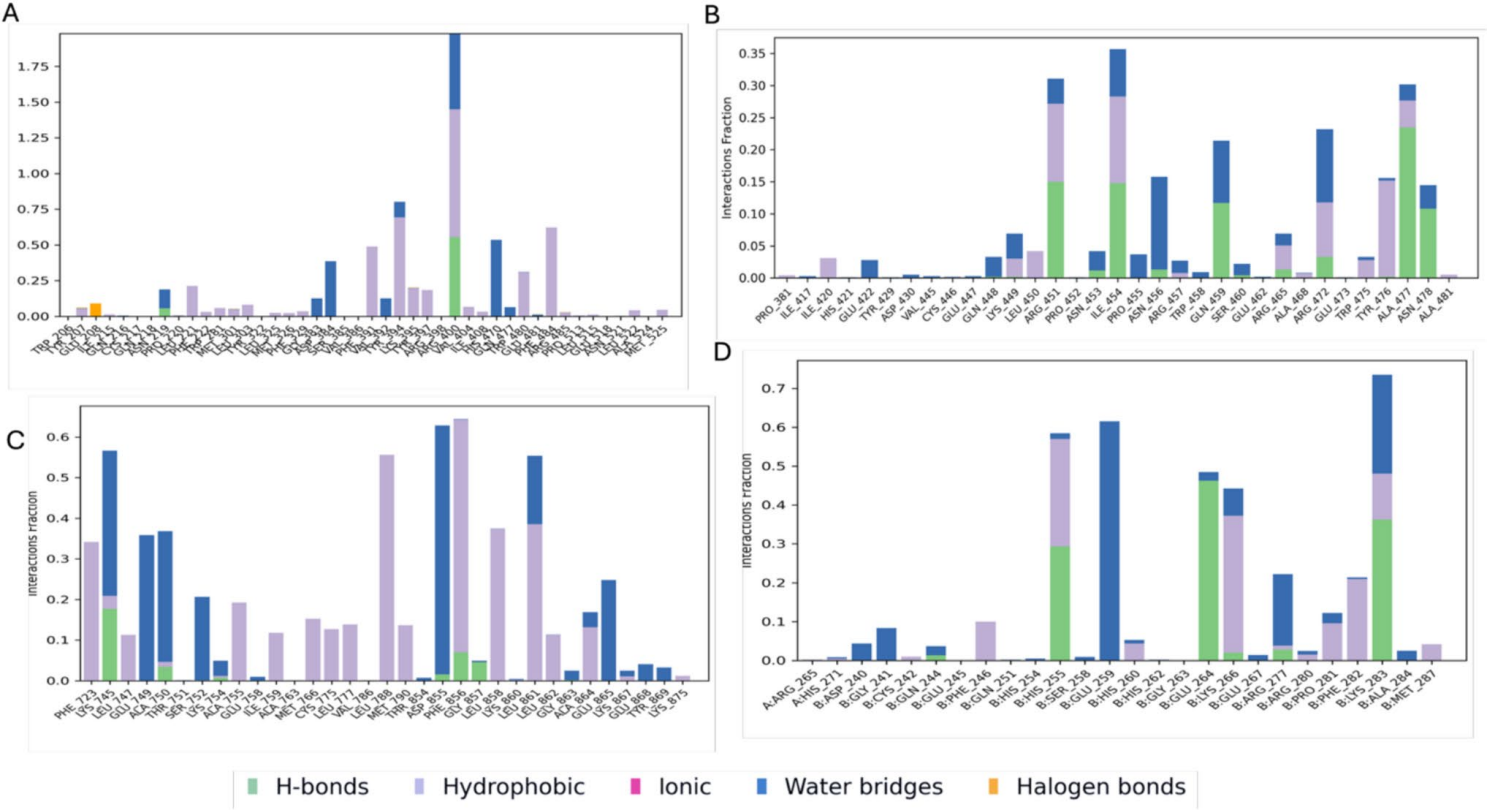
Contact histograms of Itraconazole interactions (**A**) Smoothened (5L7I), (**B**) TGF-R1 (5E8S), (**C**) EGFR (6DUK), (**D**) GLI1 (7T91). Colors: hydrophobic (green), H-bonds (pink), water bridges (blue). Interaction fraction represents the proportion of simulation frames in which each interaction type was observed across the 500 ns trajectory. Values exceeding 1.0 indicate that a residue participates in multiple concurrent interaction types within a single frame

The contact histograms (Fig. 3) illustrate the fraction of simulation time specific amino acid residues interacted with Itraconazole, categorized by interaction type (e.g., hydrogen bonds, hydrophobic, water bridges). These plots identify persistent interactions crucial for binding stability over the 500 ns MD trajectory, offering a dynamic view that complements the static docking pose.

- Smoothened (A): The interaction profile was dominated by extensive hydrophobic contacts and water bridges. Notably, Arg 400 exhibited a high aggregate interaction fraction (exceeding 1.5), reflecting concurrent contributions from hydrogen bonds (~ 0.3 fraction), water bridges, and hydrophobic contacts within individual simulation frames — consistent with its identification as a key binding residue in the initial docking pose. This dynamic profile suggests a stable binding mode dominated by hydrophobic packing and key water-mediated interactions, consistent with the stable MM/GBSA profile.
- TGF-βR1 (B): Interactions were primarily hydrophobic and hydrogen bonds within the kinase hinge region. Ala 281 and Tyr 284 showed the most persistent hydrophobic interactions (fractions > 0.3). Several residues formed transient hydrogen bonds (e.g., Lys 234, Asp 352) and water bridges, but none maintained occupancy > 50%. The relatively lower interaction fractions compared to SMO align with the less favorable MM/GBSA score, despite the high structural stability (low RMSD).
- EGFR (C): Key interactions identified in docking, like the hydrogen bond to Lys 745, were not persistent during the simulation (< 0.1 fraction). Instead, the profile was dominated by strong hydrophobic contacts with Met 766 (fraction close to 1.0) and significant interactions (> 0.5 fraction) with Leu 718 and Leu 788. While these hydrophobic interactions are strong, the lack of stable polar contacts and the high protein RMSD suggest a potentially unstable or non-specific binding mode.
- GLI1 (D): The simulation revealed extremely persistent hydrogen bonds with Gln 244 and particularly Lys 283 (fraction > 1.0), which were also identified in the docking. Hydrophobic interactions with Phe 287 were also significant (~ 0.4 fraction). However, despite these strong individual contacts within the zinc finger domain, the overall protein structure exhibited significant instability (high RMSD), suggesting these interactions are insufficient to maintain a stable complex.

### Thermal MM/GBSA

**Fig. 4:**
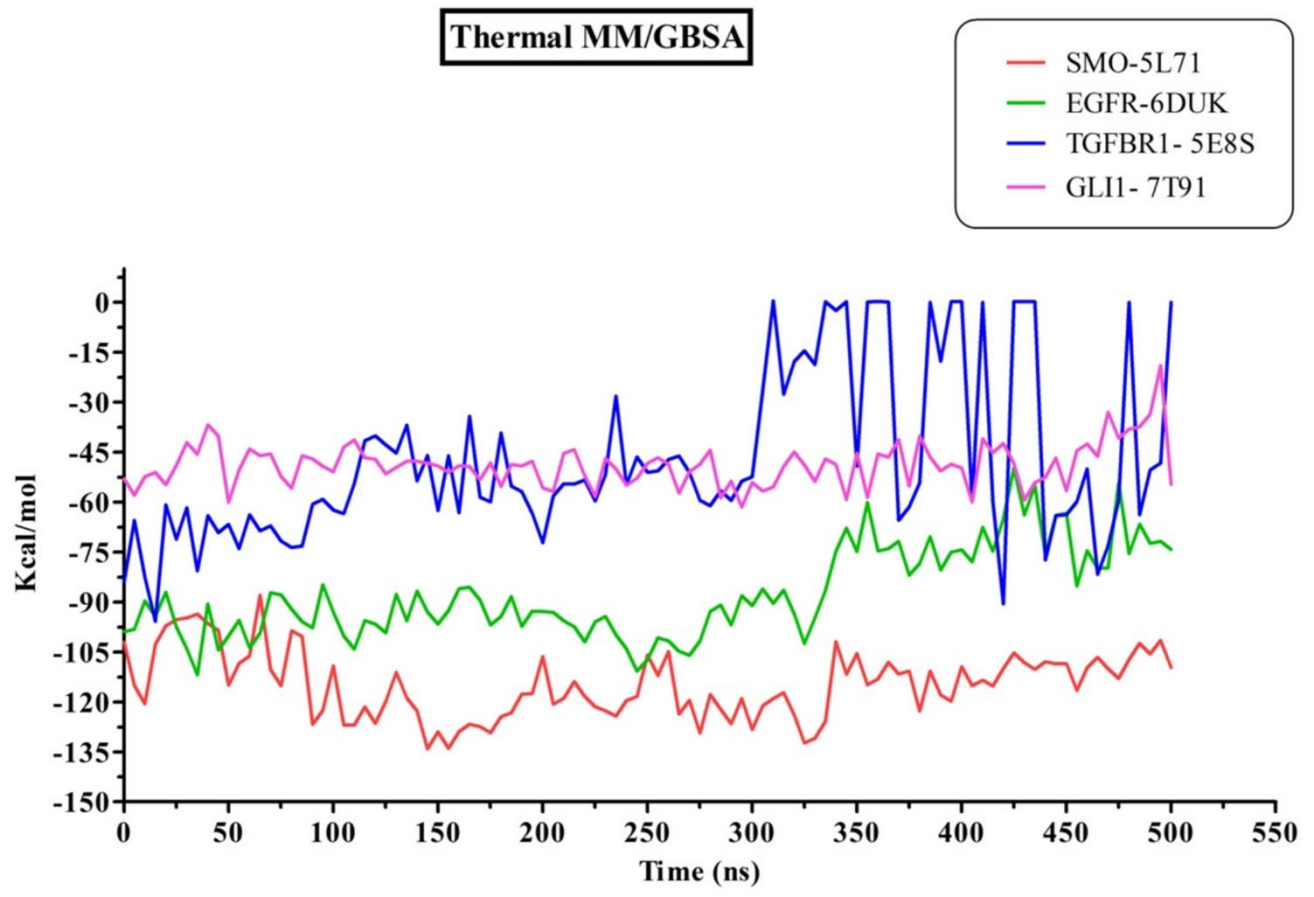
Thermal MM/GBSA profiles of the ligand‒protein complexes after 500 ns. Energy profiles of Itraconazole with each protein across the 500 ns trajectory, generated using GraphPad Prism v8.4

The Thermal MM/GBSA plot (Fig. 4) illustrates the thermodynamic favorability of the Itraconazole-protein complexes over 500 ns, with lower values indicating more stable binding. The Smoothened (SMO-5L7I) complex (red line) exhibited the most favorable and stable profile, consistently maintaining highly negative binding energies between approximately −90 and −135 kcal/mol throughout the simulation (average ΔG ≈ −104 kcal/mol). The EGFR (6DUK) complex (green line) showed moderate thermodynamic favourability initially but exhibited a progressively less favourable and non-converging profile over the trajectory, with energy values ranging from − 60 to − 100 kcal/mol (average ΔGBind ≈ − 85 kcal/mol). The failure of this profile to converge, combined with the high protein RMSD observed for this complex, undermines the reliability of this thermodynamic estimate and supports the exclusion of EGFR as a viable target. The TGF-βR1 (5E8S) complex (blue line) displayed significantly weaker and highly fluctuating binding energies, spanning a wide range from approximately 0 to −80 kcal/mol (average ΔG ≈ −45 kcal/mol), suggesting inconsistent and less favorable thermodynamic stability. The GLI1 (7T91) complex (pink line) consistently showed the least favorable binding profile, with energies typically remaining between −30 and −60 kcal/mol (average ΔG ≈ −48 kcal/mol), indicating poor thermodynamic interaction. Taking together, the convergence and stability of the MM-GBSA profiles differ markedly across the four complexes. The SMO and TGF-βR1 complexes showed converging, stable profiles consistent with their low RMSD trajectories, supporting their identification as the primary viable targets. The EGFR profile, despite a numerically moderate average, failed to converge a consequence of the conformational instability of the complex and should be interpreted with caution. The GLI1 profile, while converged, reflected the weakest absolute binding affinity.

### Control MM/GBSA benchmarking

To contextualise the Itraconazole MM/GBSA values and address the inherent limitations of cross-target thermodynamic comparisons, control 500 ns MD simulations were performed with established inhibitors for three of the four targets, using an identical protocol (100 frames, one per 5 ns). Table 3 summarises the mean ΔGBind values for both Itraconazole and its corresponding control inhibitor at each target. At SMO, Itraconazole (avg. − 104 kcal/mol) yielded a substantially more favourable mean ΔGBind than the FDA-approved inhibitor Vismodegib (− 83.20 ± 5.98 kcal/mol), consistent with published evidence of its potent anti-Hedgehog activity and reinforcing the identification of SMO as the primary viable target. At TGF-βR1, Itraconazole (avg. − 45 kcal/mol) also outperformed Galunisertib (− 29.75 ± 4.66 kcal/mol, well-converged), providing internal benchmarking support for TGF-βR1 as a viable secondary target. For EGFR, the Gefitinib control simulation run in the same 6DUK allosteric pocket also failed to converge (− 39.26 ± 17.30 kcal/mol; first/second half means − 47.72/− 30.97 kcal/mol), independently validating the instability of the EGFR allosteric pocket as a productive binding site for small molecules under these simulation conditions. For GLI1, no structurally validated inhibitor is available for the 7T91 Zif1-2 pocket; as GLI1 was excluded as non-viable on independent BPMD grounds, this does not affect the primary conclusions. Full convergence profiles for all control trajectories are presented in Fig. 6.

**Table 3:** Comparative thermal MM/GBSA binding free energies — Itraconazole vs. known inhibitors (500 ns, 100 frames). ΔGBind values calculated by thermal MM/GBSA (Prime, Schrödinger). mean and SD reported across 100 trajectory frames (1 per 5 ns). convergence assessed by comparing first- and second-half trajectory means

Target (PDB)	Ligand	Mean ΔGBind ± SD (kcal/mol)	Profile convergence
SMO (5L7I)	Itraconazole	− 104 (avg.)	Stable
SMO (5L7I)	Vismodegib (control)	− 83.20 ± 5.98	Moderate
TGF-βR1 (5E8S)	Itraconazole	− 45 (avg.)	Stable
TGF-βR1 (5E8S)	Galunisertib (control)	− 29.75 ± 4.66	Converged
EGFR (6DUK)	Itraconazole	− 85 (avg., non-converged)	Not converged
EGFR (6DUK)	Gefitinib (control)	− 39.26 ± 17.30	Not converged
GLI1 (7T91)	Itraconazole	− 48 (avg.)	Converged
*GLI1 (7T91)*	*No validated inhibitor available*	*N/A*	*N/A*

### Binding pose metadynamics (BPMD)

**Fig. 5:**
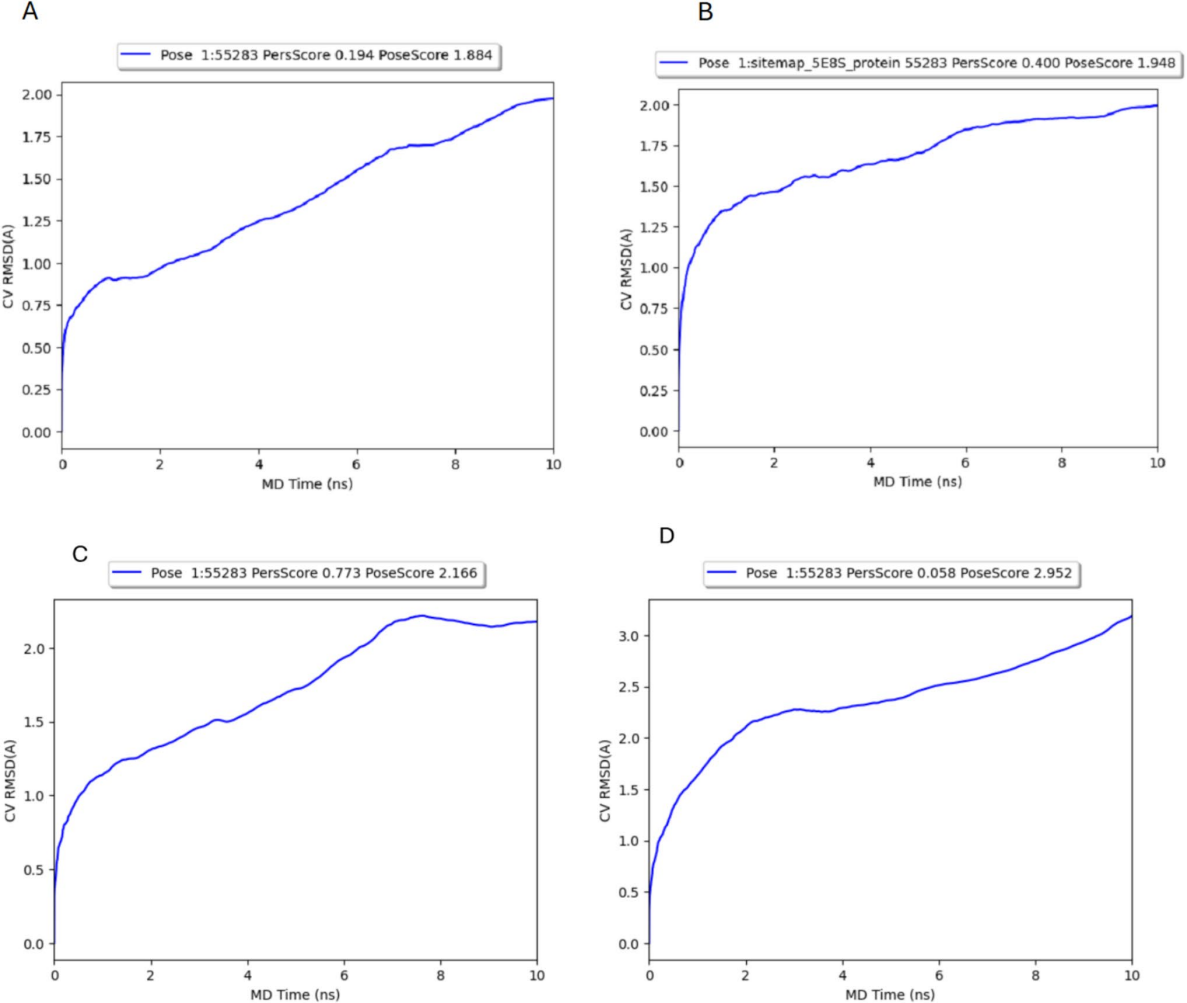
BPMD profiles of Itraconazole against the four proteins: **A**) Smoothened; **B**) TGFβR1; **C**) EGFR; **D**) GLI1.CV RMSD: collective variable root mean square deviation, measuring the positional deviation of key pharmacophoric atoms of the ligand from the reference docked pose over the simulation trajectory

The binding pose metadynamics analysis (Fig. 5) estimates the stability of the ligand's binding pose by calculating the free energy required to displace it from the binding site (PoseScore, related to ΔG of unbinding) and its persistence in that pose over the simulation time (PersScore). Lower PoseScores indicate more stable poses. The analysis revealed that Itraconazole binding to TGF-βR1 (5E8S, PoseScore 1.99 kcal/mol) and SMO (5L71, PoseScore 1.97 kcal/mol) exhibited the strongest pose stability, suggesting high ligand retention within these binding sites. EGFR (6DUK, PoseScore 2.18 kcal/mol) showed moderate pose stability, despite having a high persistence score (0.773), which likely reflects the strong hydrophobic contacts observed. GLI1 (7T91) displayed the least stable pose (PoseScore 3.19 kcal/mol) and very low persistence (0.058), indicating poor ligand retention. Overall, BPMD suggests that Itraconazole forms the most stable binding poses with SMO and TGF-βR1.

## Discussion

The inherent capabilities of EMT in breast cancer make it a concerning event in the progression of BC. The literature suggests that targeting EMT in breast cancer patients will likely lead to improved life expectancy and clinical outcomes. Although the signaling pathways that are involved in EMT are well studied, targeting proteins has become a challenging task. In this study, Itraconazole was identified as a potential drug repurposing candidate for the purpose of inhibiting EMT in breast cancer. Virtual docking has become an important tool for understanding the interactions of molecules. It helps in quickly analyzing the interaction profiles, with the only limitation being the availability of the solved three-dimensional structure of the protein of interest. In particular, when combined with the induced fit protocol, MD and post-MD thermodynamic calculations virtual screening will help to a greater extent.

A critical analysis of our data reveals apparent discrepancies between the computational methods, which highlights the distinct properties each method evaluates. IFD provides a static score of the initial pose, while Protein RMSD measures the structural stability of the entire complex, and MM/GBSA calculates the thermodynamic favorability (ΔG) of binding over the simulation. BPMD complements this by assessing the free energy barrier to unbinding (pose stability).

Based on a balanced assessment of these methods, SMO emerges as the clear primary target. It not only achieved the best IFD score (−15.405 kcal/mol) but also demonstrated the most favorable and stable thermodynamic profile in the MM/GBSA analysis (avg. −104 kcal/mol). While its protein RMSD showed an initial adjustment period before stabilizing ~ 3.0 Å (Fig. 2A), this is indicative of a classic 'induced fit’ a re-orientation confirmed by the dynamic contact histogram (Fig. 3A) rather than instability. This strong thermodynamic binding to the Vismodegib pocket, consistent with published literature [15, 17–20], makes it the most promising target.

The case for TGF-βR1 as a viable target is more complex. The complex showed the highest structural stability (Protein RMSD < 2.25 Å) and excellent pose stability (BPMD 1.99 kcal/mol), confirming Itraconazole can form a stable complex in the kinase hinge region. However, this is contrasted by its poor IFD score and, more importantly, the highly fluctuating and significantly less favorable MM/GBSA profile (avg. −45 kcal/mol). This common discrepancy implies that while Itraconazole can form a structurally stable pose, its thermodynamic binding is weaker and less consistent than with SMO. Therefore, we posit that SMO is the primary target, while TGF-βR1 is a plausible, but secondary, target.

Conversely, EGFR and GLI1 were assessed as non-viable targets, though for distinct reasons. For EGFR, the initial docking score was competitive (− 12.998 kcal/mol, comparable to Gefitinib at − 13.065 kcal/mol), and the mean MM-GBSA value was numerically moderate (avg. − 85 kcal/mol). However, the EGFR complex exhibited significant and persistent conformational instability throughout the 500 ns trajectory, and the MM-GBSA profile failed to converge a direct consequence of this instability. A non-converged thermodynamic profile cannot be interpreted as a reliable binding free energy estimate regardless of its numerical mean. Furthermore, the allosteric pocket in 6DUK, while structurally accessible in the inactive conformation, did not sustain productive ligand retention under dynamic simulation conditions, as confirmed by the BPMD pose score (2.18 kcal/mol) and the near-absent persistence of the key Lys 745 hydrogen bond (< 0.1 fraction). For GLI1, the complex exhibited a transient conformational excursion reaching ~ 4.2 Å RMSD at 350 ns before recovering to baseline (~ 2 Å), suggesting conformational flexibility rather than irreversible destabilisation. However, the BPMD analysis revealed the weakest pose stability across all four targets (PoseScore 3.19 kcal/mol, PersScore 0.058), indicating poor ligand retention inconsistent with viable target engagement, supported by the weakest thermodynamic profile (avg. − 48 kcal/mol).

**Fig. 6:**
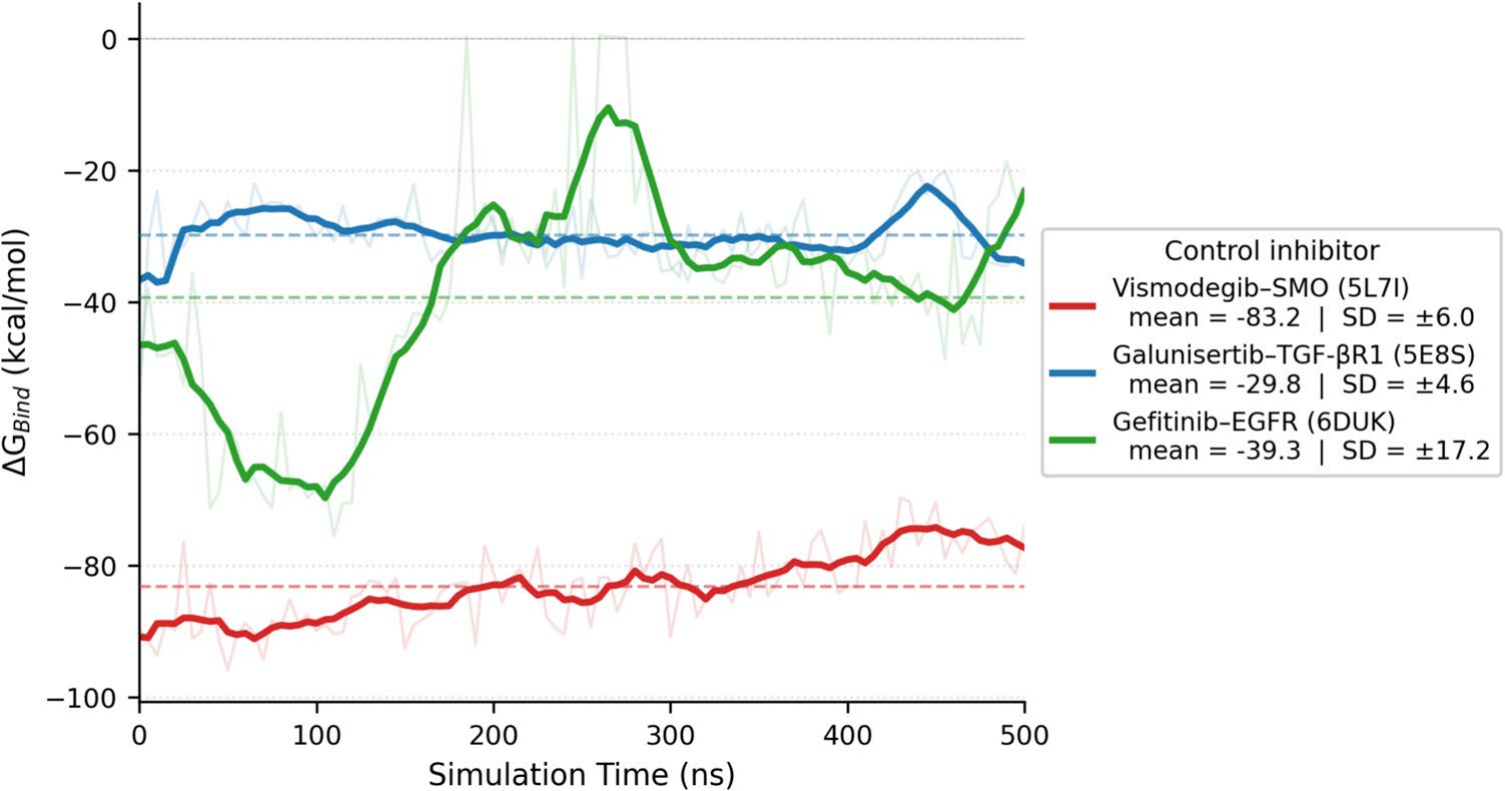
Thermal MM/GBSA binding free energy profiles of known inhibitor controls over 500 ns. Vismodegib–SMO (red), Galunisertib–TGF-βR1 (blue), and Gefitinib–EGFR (green) are overlaid. transparent lines = raw frame values; solid lines = smoothed profiles (uniform filter, window = 8 frames); dashed lines = trajectory mean. Shaded band = mean ± 1 SD. Mean ΔGBind: Vismodegib–SMO − 83.20 ± 5.98 kcal/mol (moderately converged); Galunisertib–TGF-βR1 − 29.75 ± 4.66 kcal/mol (converged); Gefitinib–EGFR − 39.26 ± 17.30 kcal/mol (non-converged)

The internal benchmarking provided by the control MD simulations (Fig. 6, Table 3) strengthens the selectivity conclusions of this study. At SMO, Itraconazole (avg. − 104 kcal/mol) demonstrated substantially more favourable thermodynamic binding than the FDA-approved inhibitor Vismodegib (− 83.20 kcal/mol), consistent with published biochemical evidence of its potent anti-Hedgehog activity and its ability to inhibit Vismodegib-resistant SMO variants through a distinct mechanism. At TGF-βR1, Itraconazole (avg. − 45 kcal/mol) similarly outperformed Galunisertib (− 29.75 kcal/mol), and the Galunisertib profile was well-converged (drift 0.93 kcal/mol), confirming the reliability of the TGF-βR1 binding free energy estimates. Most notably, the Gefitinib control at the EGFR allosteric pocket (6DUK) was itself non-converged (− 39.26 ± 17.30 kcal/mol; drift 16.75 kcal/mol), providing independent structural evidence that the EGFR allosteric pocket does not productively retain small molecule ligands under these simulation conditions, regardless of their initial docking affinity. This result, taken together with the Itraconazole-EGFR instability data, elevates the EGFR exclusion from a single-ligand observation to a pocket-level finding, substantially strengthening the selectivity argument for SMO and TGF-βR1 as the primary viable targets.

This dual-target anti-cancer activity is not a class effect shared by all azole antifungals. Simpler azoles, such as Fluconazole or Voriconazole, lack the extensive and complex lipophilic side chains that define Itraconazole's structure. It is this unique structure that enables Itraconazole to engage in the specific, stable interactions with allosteric sites on SMO and the TGF-βR1 kinase domain, as demonstrated in our simulations, an activity distinct from its primary antifungal mechanism. However, from a translational perspective, these in silico findings must also be weighed against Itraconazole's known pharmacokinetic challenges. Its high lipophilicity, poor aqueous solubility, variable oral bioavailability, and extensive plasma protein binding (~ 99.8%) are critical hurdles that will influence the achievable free-drug concentration at the tumor site and must be accounted for in future in vitro and in vivo models.

The most promising clinical translation potential for Itraconazole, based on our findings, likely lies in its use as part of a combination therapy. EMT, driven by pathways like Hedgehog (via SMO) and TGF-β, is a primary mechanism of acquired resistance to standard chemotherapeutics (e.g., taxanes) in breast cancer. Our in-silico data provide a strong molecular rationale for a strategy where Itraconazole is used as an "EMT-reversing" or "chemo-sensitizing" agent. By dually inhibiting both SMO and TGF-βR1, Itraconazole could potentially dismantle the EMT program, making resistant mesenchymal-like cells revert to an epithelial state that is once again susceptible to conventional chemotherapy. This suggests a direct and testable in vitro hypothesis: co-administration of Itraconazole with a drug like paclitaxel should show synergistic, not just additive, anti-tumor effects in resistant breast cancer cell lines, a hypothesis supported by existing preclinical evidence of Itraconazole's synergism with paclitaxel in epithelial ovarian cancer [21], its synergistic G0/G1 cell cycle arrest with rapamycin in TNBC cell lines [22], and improved clinical outcomes in heavily pre-treated TNBC patients receiving Itraconazole combined with docetaxel-based regimens [23].

Control MD simulations with known inhibitors were performed for three of the four targets (Vismodegib–SMO, Galunisertib–TGF-βR1, Gefitinib–EGFR) and are presented in the Results section (Table 3, Fig. 6). These simulations demonstrate that Itraconazole compares favourably to both Vismodegib and Galunisertib at their respective targets, and that the EGFR allosteric pocket is inherently unstable for small molecule binding under these simulation conditions, as confirmed by the Gefitinib control. A parallel control simulation for GLI1 was not possible as no structurally validated inhibitor is available for the 7T91 Zif1-2 pocket. Furthermore, with respect to Smoothened, our study was limited to evaluating Itraconazole’s binding only within the canonical Vismodegib pocket. The evidence that Itraconazole may inhibit Vismodegib-resistant signaling [24] strongly suggests its mechanism could involve alternative allosteric sites, which remains a critical area for future investigation. Finally, although our data are supported by extensive 500 ns of molecular dynamics simulations and favorable binding energy profiles, they must be verified with in vitro cell culture and molecular biology experimental data.

Future computational work should also move beyond single-point comparisons. To better understand the chemical features driving Itraconazole's selectivity for SMO and TGF-βR1 over EGFR and GLI1, pharmacophore modeling and 3D molecular fingerprint analysis would be highly valuable. These methods could generate a selectivity model, guiding the design of new Itraconazole analogs with improved potency and a more tailored multi-target profile.

## Materials and methods

### Structure preparation

Protein structures were retrieved from the Protein Data Bank (PDB-https://www.rcsb.org) [25]. All the PDB identifiers are listed in Table 4. The 3D structure of Itraconazole was retrieved from PubChem (PubChem ID 55283) [26]. The Itraconazole structure was prepared for docking via the LigPrep tool of Schrodinger [27]. Protein Preparation Wizard was used to optimize the protein structures for docking[28]. The optimization parameters included the modulation of water molecules and hydrogen bonds and the filling of missing loops and side chains.

**Table 4:** Proteins shortlisted for docking, their protein data bank IDs and rationale for selection [32–35]

Sr No	Protein	PDB ID	Rationale
1	Smoothened (SMO)	5L7I	SMO is the canonical downstream effector of Patched (PTCH) in the Hedgehog signalling pathway, responsible for activating GLI transcription factors that drive pro-EMT gene expression [16]. Itraconazole has been directly demonstrated to inhibit SMO via prevention of its ciliary accumulation through a mechanism distinct from Vismodegib, conferring activity even against Vismodegib-resistant SMO variants [15]. This published mechanistic evidence makes SMO the primary rationale for evaluating Itraconazole as a computational EMT inhibitor candidate
2	TGF-βR1	5E8S	TGFβR1 is the type I receptor of the TGF-β signalling axis — the most extensively characterised EMT-inducing pathway in breast cancer — which upon activation drives expression of pro-EMT transcription factors Snail, ZEB1, and Twist via SMAD-dependent and non-canonical pathways [8]. Itraconazole has been demonstrated to inhibit TGF-β/SMAD2/3 signalling, suppress EMT markers, and reduce invasion and migration in pancreatic cancer cells, providing direct mechanistic precedent for its evaluation at this target [36]. This evidence positions TGFβR1 as a biologically motivated secondary target for investigation
3	EGFR	6DUK	EGFR signalling drives EMT through activation of Ras/MAPK and PI3K/AKT pathways, conferring invasiveness, metastatic potential, and poor clinical outcomes particularly in triple-negative breast cancer [13, 14]. PDB 6DUK captures EGFR in the inactive αC-helix-out conformation, which uniquely exposes the allosteric binding pocket targeted by inhibitors such as EAI045 [16]. This structure was selected to test whether Itraconazole, which acts allosterically at SMO, could similarly engage the EGFR allosteric site — a hypothesis evaluated and resolved by the MD simulation data
4	GLI-1	7T91	GLI1 is the zinc finger transcription factor at the terminal end of Hedgehog signalling, downstream of SMO, responsible for driving expression of MDR-1 and VEGFR2 — mediators of drug resistance and angiogenesis in breast cancer [11, 12]. Including GLI1 alongside SMO allows evaluation of whether Itraconazole acts at multiple nodes of the Hedgehog-EMT axis simultaneously. PDB 7T91 captures the apo linearized homodimer conformation of GLI1 Zif1-2, exposing a hydrophobic interface that represents a structurally validated druggable pocket distinct from the DNA-bound GLI1 conformation [37]

### Grid generation

Mapping of the active site and grid for docking was based on cocrystallized ligands. In the case of nonavailability of co-crystalized ligands, the sitemap tool was used to identify the potential binding site [29]. The Glide Grid tool was used to designate the grid for docking.

### Induced fit docking

Induced Fit docking (IFD) was performed via the Schrödinger IFD program with extensive sampling of 80 replicas per pose[30]. The docking score was used to rank the ligands and their poses. Ligand interactions were recorded via a ligand interaction diagram.

### Molecular dynamics

Molecular dynamics simulations were performed to further analyze the interactions and structures of the proteins [31].

#### System building

Following IFD, poses were ranked by IFD score, a composite of GlideScore and the Prime energy of the receptor-ligand complex. The top-ranked pose was further evaluated by visual inspection in Maestro for accommodation within the binding site, conservation of key pharmacophoric interactions with functionally important residues, and absence of steric clashes. The highest-scoring pose satisfying these criteria was selected to initiate MD simulation using the Desmond module. The system builder tool of Desmond was used for building the system for molecular dynamics with default parameters, and we used Na^+^ or Cl^−^ ions to neutralize the system during the system-building step ^26^ [31].

#### System relaxation

We have performed a standard six-step relaxation protocol to ensure the system was properly minimized and equilibrated:

- Minimization: Steepest descent minimization was performed with position restraints on solute heavy atoms to a maximum convergence criterion of 0.5 kcal/mol/Angstrom.
- Solvent Relaxation (NVT): A short MD simulation (12 ps) was run in the NVT ensemble (T = 10 K) with restraints on solute heavy atoms, allowing the solvent to relax around the fixed solute.
- Heating (NVT): The system was heated to 300 K over 12 ps in the NVT ensemble with small restraints (20 kcal/mol/Angstrom2) applied to solute heavy atoms.
- Initial Equilibration (NPT): A short NPT ensemble simulation (12 ps) was performed at 300 K and 1.0 bar with restraints still applied.
- Final Equilibration (NPT): A final, longer NPT ensemble simulation (100 ps) was run at 300 K and 1.0 bar with all restraints removed.

#### Production MDs

We performed 500 ns of MD with default parameters unless otherwise specified. The RMSD (Fig. 2) and contact histograms (Fig. 3) were extracted via the Simulation Interaction Diagram of the Schrödinger Suite. Water bridge interactions were identified using the SID module, defined as contacts where a bridging water molecule simultaneously hydrogen-bonds to both the ligand and a protein residue, with a distance cutoff of 2.5 Å and a donor-hydrogen-acceptor angle cutoff of 120°.

#### MM-GBSA binding free energy calculations

Binding free energies (ΔGBind) were calculated using the Thermal MM-GBSA script within the Schrödinger Suite, applied across the full 500 ns MD trajectory. One hundred evenly spaced frames were extracted (one frame every 5 ns) for calculation. The binding free energy was decomposed as

$$\Delta {G}_{Bind}=\Delta {E}_{MM}-\Delta {G}_{Solv}-T\Delta S$$

where ΔEMM is the change in molecular mechanics energy in the gas phase (van der Waals and electrostatic contributions); ΔGSolv is the change in solvation free energy comprising the polar term (ΔGGB, generalised Born model) and non-polar term (ΔGSA, solvent-accessible surface area); and TΔS is the conformational entropy contribution calculated using normal mode analysis as implemented in the Prime module of Schrödinger. The resulting ΔGBind values were plotted as a function of simulation time (Fig. 4) to assess convergence of the thermodynamic estimate over the trajectory.

#### Binding pose metadynamics (BPMD)

Binding Pose Metadynamics (BPMD) simulations were performed using Schrodinger Suite to evaluate the stability of the ligand within the active site of the target protein. Each simulation was performed for 10 replicas of 10 ns each and the data was plotted RMSD in Angstroms as CV (Y-axis) for pose stability vs simulation time (X-axis).

## Data Availability

Additional data can be shared upon request by contacting the corresponding author, Jesil Mathew Aranjani (jesil.m@manipal.edu).
